# 887 Inhalation Injuries Evaluated on a Global Scale

**DOI:** 10.1093/jbcr/iraf019.418

**Published:** 2025-04-01

**Authors:** Daniel Najafali, Megan Najafali, Hilary Liu, Saeid Rezaei, José Arellano, Logan Galbraith, Mare Kaulakis, Christopher Fedor, Francesco Egro

**Affiliations:** Carle Illinois College of Medicine; Loyola University Chicago Stritch School of Medicine; University of Pittsburgh Medical Center; University of Pittsburgh Medical Center; University of Pittsburgh Medical Center; Northeast Ohio Medical University; University of Pittsburgh School of Medicine; University of Pittsburgh School of Medicine; University of Pittsburgh Medical Center

## Abstract

**Introduction:**

Inhalation injuries pose serious harm to individuals sustaining burn injuries. Despite the severity of inhalation injuries, data on their global incidence, treatment patterns, and outcomes—especially in low-resource settings—are limited. This study aims to analyze the global impact of inhalation injuries and quantify their influence on mortality.

**Methods:**

Entries from the WHO Global Burn Registry were stratified based on whether or not patients had inhalation injuries. Descriptive statistics for each cohort were presented for demographics, burn characteristics, facility characteristics, and disposition. Multivariable regression analysis quantified the influence of inhalation injury on mortality and the need for surgical intervention.

**Results:**

There were 1,382 cases of inhalation injury. Inhalation injury patients had significantly higher median TBSA (50% vs. 15%, P< 0.001), consisted of flame-based injuries (89%), were significantly older (P< 0.001), and were mostly males (54%). Inhalation injury patients underwent surgery at a significantly lower rate (34% vs. 55%, P< 0.001). A total of 63% of inhalation injury patients died due to their injuries. Multivariable logistic regressions demonstrated that inhalation injury significantly increased the odds of mortality (OR 2.65, 95%CI 2.16-3.25) and reduced the odds of surgical intervention (OR 0.58, 95%CI 0.50-0.66).

**Conclusions:**

Patients with inhalation injuries are particularly susceptible to worse outcomes. Additional factors, such as the timing of tracheostomies should be explored as a modifiable factor that could improve patient outcomes.

**Applicability of Research to Practice:**

The global burn community must prioritize the management of inhalation injuries, particularly in resource-limited settings. Enhancing critical care capacities, particularly access to ventilators, could improve survival rates in this vulnerable patient population.

**Funding for the Study:**

N/A

